# IL-8 as a Potential Therapeutic Target for Periodontitis and Its Inhibition by Caffeic Acid Phenethyl Ester In Vitro

**DOI:** 10.3390/ijms22073641

**Published:** 2021-03-31

**Authors:** Yung-Kai Huang, Kuo-Feng Tseng, Ping-Hsuan Tsai, Jie-Sian Wang, Chang-Yu Lee, Ming-Yi Shen

**Affiliations:** 1 Department of Oral Hygiene, College of Dental Medicine, Kaohsiung Medical University, Kaohsiung 80708, Taiwan; ykhuang@kmu.edu.tw; 2 Department of Biological Sciences and Technology, China Medical University, Taichung 40402, Taiwan; u107010409@cmu.edu.tw; 3 Graduate Institute of Biomedical Sciences, College of Medicine, China Medical University, Taichung 40402, Taiwan; U105010312@cmu.edu.tw (P.-H.T.); d29745@mail.cmuh.org.tw (J.-S.W.); 4 Division of Nephrology, Department of Internal Medicine, China Medical University Hospital, Taichung 40402, Taiwan; 5 Department of Dentistry, Taipei Medical University Hospital, Taipei 110301, Taiwan; m8504005@tmu.edu.tw; 6 Department of Medical Research, China Medical University Hospital, Taichung 40402, Taiwan; 7 Department of Nursing, Asia University, Taichung 41354, Taiwan

**Keywords:** interleukin-8, oral microbiota, caffeic acid phenethyl ester, periodontitis, THP-1 cell

## Abstract

Salivary levels of interleukin-8 (IL-8) are elevated in patients with periodontitis. Caffeic acid phenethyl ester (CAPE) improves the periodontal status in subjects. However, whether CAPE can reduce IL-8 expression is unclear. We collected saliva to determine proinflammatory cytokine levels and used subgingival calculus and surrounding tissues from patients with periodontitis for oral microbiota analysis via 16s ribosomal RNA gene sequencing. THP-1 cells were stimulated with sterile-filtered saliva from patients, and target gene/protein expression was assessed. IL-8 mRNA expression was analyzed in saliva-stimulated THP-1 cells treated with CAPE and the heme oxygenase-1 (HO-1) inhibitor tin-protoporphyrin (SnPP). In 72 symptomatic individuals, IL-8 was correlated with periodontal inflammation (bleeding on probing, *r* = 0.45; *p* < 0.001) and disease severity (bleeding on probing, *r* = 0.45; *p* < 0.001) but not with the four oral microbiota species tested. Reduced salivary IL-8 secretion was correlated with effective periodontitis treatment (*r* = 0.37, *p* = 0.0013). In THP-1 cells, saliva treatment induced high IL-8 expression and IKK2 and nuclear factor-κB (NF-κB) phosphorylation. However, the IKK inhibitor BMS-345541, NF-κB inhibitor BAY 11-7082, and CAPE attenuated saliva-induced IL-8 expression. CAPE induced HO-1 expression and inhibited IKK2, IκBα, and NF-κB phosphorylation. Blocking HO-1 decreased the anti-inflammatory activity of CAPE. The targeted suppression of IL-8 production using CAPE reduces inflammation and periodontitis.

## 1. Introduction

Nearly 50% of the world’s population suffers from periodontal disease. Studies have shown that periodontitis is correlated with systemic disorders, including diabetes, cardiovascular disease, endocrine disorders, and rheumatoid arthritis. The high prevalence of periodontal disease has made it a worldwide burden [1,2,3]. Periodontitis is an inflammatory disease primarily characterized by a periodontal infection-induced immune imbalance, which leads to inflammation of the periodontal soft tissue, eventually resulting in symptoms such as periodontal ligament damage and alveolar bone loss [4]. Upon damage to the alveolar bone and periodontal tissue, which are surrounded by the gums, gap formation begins between the teeth and gums. These gaps lead to formation of periodontal pockets, exacerbating periodontal ligament damage and alveolar bone loss. Consequently, the probing depth of periodontal pockets increases with bone loss. Clinically, the plaque index (PI), bleeding on probing (BOP), and probing pocket depth (PPD) are used as parameters to reflect disease activity. A decrease in or recovery of the pocket depth can be used as an indicator of treatment efficacy. The dynamic development of disease activity can be measured based on tissue degradation and bone resorption, as well as by using biomarkers, such as inflammatory and antioxidant markers present in the patients’ blood, saliva, and gingival crevicular fluid [5]. Effective biomarkers can be used as early predictive indicators of clinical disease, facilitating the assessment of disease onset or recovery in individuals [6].

Nuclear factor-κB (NF-κB) is an important transcription factor involved in the inflammation process. After activation, NF-κB translocates into the nucleus, where it binds to proinflammatory genes and induces the formation of proinflammatory cytokines such as interleukin (IL)-1α, IL-1β, IL-6, IL-8, and tumor necrosis factor-α (TNF-α) [7]. When periodontal tissue becomes inflamed, an increase in the concentrations of proinflammatory cytokines, IL-1, IL-6, and TNF-α, which are the markers of systemic inflammation, is observed. Subsequently, lymphatic and myeloid cells are induced to secrete matrix metalloproteinases, which are involved in the metabolism of periodontal tissue and decomposition of the extracellular matrix. When IL-8 and TNF-α are activated, the number and activity of polymorphonucleocytes simultaneously increase. Phagocytosis leads to intracellular toxicity and the subsequent release of oxygen radicals; moreover, lipid peroxides and oxidized proteins produced by the periodontal tissue further damage the activated macrophages and generate more reactive oxygen species [8,9]. In addition, heme oxygenase-1 (HO-1), which plays a role in orthodontic tooth movement and inflammation in periodontitis models [10], might activate cellular defense mechanisms against oxidative stress [11].

Proinflammatory factors present in the saliva, such as IL-1, IL-6, IL-8, TNF-α, as well as the inflammation marker matrix metalloproteinase-8, have been confirmed to reflect systemic inflammation [12]. In addition to their use in disease prevention, biomarkers can be used to predict treatment efficacy. *Streptococcus salivarius* induces the gene expression of IL-6 and IL-8 in oral mucosa cells [13], whereas *Porphyromonas gingivalis* stimulates IL-8 gene expression in gingival fibroblasts and periodontal ligament fibroblasts [14,15]. A recent study revealed a significant reduction in the salivary levels of proinflammatory cytokines such as IL-1β, IL-6, and IL-8 in patients who underwent non-surgical periodontal therapy [16]. However, few studies have explored the correlations between salivary cytokine biomarkers, including IL-1β, IL-6, and IL-8, and clinical disease indicators of periodontitis before and after non-surgical periodontal therapy.

Caffeic acid phenethyl ester (CAPE) is a central active component of propolis, which is obtained from honeybee hives [17]. Propolis has multiple applications in dentistry, and therefore, CAPE is of potential clinical interest [18,19,20]. The aim of the present study was to investigate whether IL-8 can serve as a therapeutic target for periodontitis, and to determine whether CAPE reduces inflammation through the suppression of IL-8 expression in THP-1 cells.

## 2. Results

### 2.1. The Impact of Proinflammatory Cytokines and the Oral Microbiota on the Severity of Periodontal Disease

We first examined the effects of proinflammatory cytokines and the oral microbiota on the severity of periodontitis. Table 1 shows the correlation coefficients between the baseline clinical parameters, i.e., the PI, BOP, and PPD, and levels of salivary proinflammatory cytokines or relative abundances of certain bacteria in the oral microbiota in patients with periodontal disease. There was a significant correlation between BOP and the levels of IL-8 (*r* = 0.45; *p* < 0.001). Positive correlations of the mean PPD with IL-8 (*r* = 0.49; *p* < 0.001) levels are also shown in Table 1. There were no significant correlations between the baseline percentages of the four bacterial species in the oral microbiota and clinical parameters. Thus, salivary IL-8 concentrations were positively correlated with the severity of periodontal disease before treatment but were not associated with the relative abundances of the four bacteria, *P. gingivalis*, *Prevotella intermedia*, *Fusobacterium nucleatum*, and *Aggregatibacter actinomycetemcomitans*.

In addition, there were no significant correlations (*r*-values: −0.19–0.09; *p*-values: 0.11–0.98) between the indicators of clinical effectiveness in patients before and after treatment as well as the proportions of the four bacteria before treatment (Table 2). These data suggest that proinflammatory cytokines have a greater impact than the oral microbiota on the severity of periodontal disease.

### 2.2. The Relationship between Oral Microbiota and Salivary Proinflammatory Cytokines

**Figure 1:**
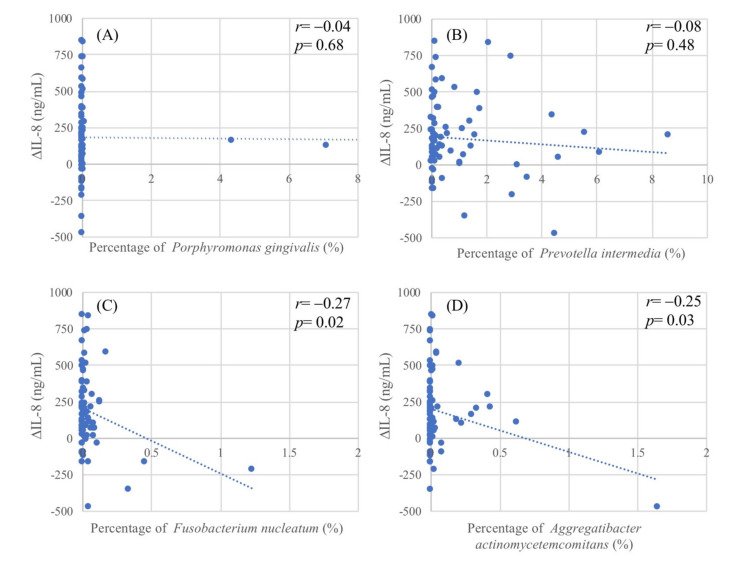
Scatter plots of differences between pre- and post-treatment levels of salivary interleukin-8 (IL-8) in patients with periodontitis versus respective percentages of representative bacterial species in the oral microbiota: (**A**) *Porphyromonas gingivalis*; (**B**) *Prevotella intermedia*; (**C**) *Fusobacterium nucleatum*; and (**D**) *Aggregatibacter actinomycetemcomitans*. ∆IL-8 (ng/mL) = baseline IL-8–post-treatment IL-8.

Next, we evaluated the potential relationship between the oral microbiota and salivary proinflammatory cytokines in patients with periodontal disease. The scatter plots (Figure 1) show the extent of reduction in salivary IL-8 (∆IL-8) levels after treatment versus the percentages of each of the bacteria in the oral microbiota. There were significant negative correlations between the percentages of *F. nucleatum* and *A. actinomycetemcomitans* and ∆IL-8, with coefficients of correlation of −0.27 (*p* = 0.02; Figure 1C) and −0.25 (*p* = 0.03; Figure 1D), respectively. There were no significant correlations between the oral microbiota and the other two salivary cytokines (IL-6 and TNF-α).

### 2.3. Contribution of IL-8 to the Treatment Outcome of Periodontal Disease

**Figure 2:**
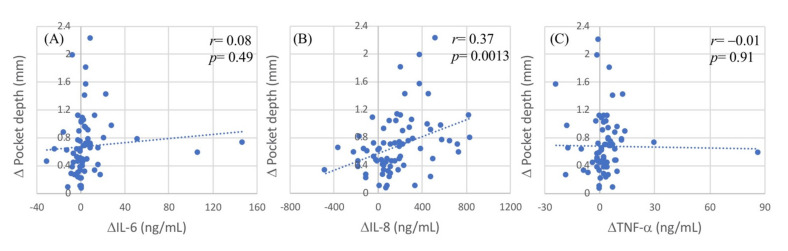
Scatter plot of difference (∆) in IL-8 and (∆) in clinical indices. (**A**) Difference (∆) in salivary interleukin-8 (ng/mL) and difference (∆) in plaque index (%). (**B**) Difference (∆) in salivary interleukin-8 (ng/mL) and difference (∆) in bleeding on probing (%). (**C**) Difference (∆) in salivary interleukin-8 (ng/mL) and difference (∆) in pocket depth (mm). ∆IL-8 = baseline IL-8–after treatment IL-8; ∆plaque index = baseline plaque index–after treatment plaque index. ∆Pocket depth = baseline pocket depth–after treatment pocket depth.

The scatter plots (Figure 2) show the extent of reduction in the effectiveness of periodontal treatment (∆PPD) values versus those in salivary cytokine concentrations after treatment. There was a significant correlation between ∆PPD and ∆IL-8 values, with correlation coefficients of 0.37 (*p* = 0.0013; Figure 2B); however, ∆PPD values were not associated with ∆IL-6 (Figure 2A) or ∆TNF-α (Figure 2C), nor was there any significant correlation between the ∆PI or ∆BOP values and any of the proinflammatory cytokines. The correlation coefficients of the reduction in oral hygiene index (∆PI) with the ∆IL-6, ∆IL-8, and ∆IL-6 values were −0.03 (*p* = 0.83), −0.13 (*p* = 0.26), and −0.11 (*p* = 0.37), respectively. The correlation coefficients of the reduction in the periodontal inflammatory index (∆BOP) with the ∆IL-6, ∆IL-8, and ∆IL-6 values were −0.08 (*p* = 0.48), 0.12 (*p* = 0.30), and −0.09 (*p* = 0.42), respectively. The data showed that the salivary IL-8 concentrations were significantly correlated with the effectiveness of periodontal treatment. A greater reduction in salivary IL-8 after treatment was associated with better effectiveness of periodontal disease treatment. Thus, reducing salivary IL-8 secretion can increase the effectiveness of periodontal disease treatment.

### 2.4. Saliva-Induced IL-8 Expression in THP-1 Cells

**Figure 3:**
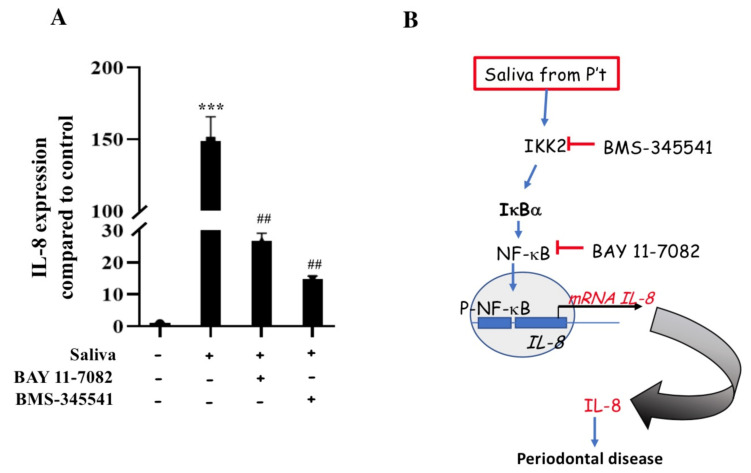
Regulation of saliva-induced interleukin-8 (IL-8) expression in THP-1 cells via the IKK2/IκBα/NF-κB pathway. (**A**) mRNA expression levels of IL-8 in cells exposed to saliva (5%) and control cells. Effects of IKK2 and NF-κB inhibitors (BMS-345541 and BAY 11-7082, respectively) on saliva-induced mRNA expression of IL-8 in THP-1 cells. Data represent the mean ± standard error of the mean (*n* = 3). *** *p* < 0.001 compared to control group; ^##^ *p* < 0.01 compared to saliva-treated group (Student’s *t*-test). (**B**) Working scheme for the regulation of saliva-induced IL-8 expression via the IKK2/IκBα/NF-κB pathway. Blue arrow = stimulation; red line with an end bar = inhibition; black arrow = direction or flow.

Based on the clinical findings, we further evaluated whether saliva from patients with periodontal disease increases IL-8 expression in human immune cells. Our data showed that the expression of IL-8 mRNA was significantly higher in THP-1 cells exposed to saliva obtained from patients with periodontal disease than that in control cells (*p* < 0.001; Figure 3A). This finding indicated the presence of IL-8 expression-promoting factors in the saliva of patients with periodontal disease (Figure 3B).

### 2.5. Induction of IL-8 Expression via the IKK2/IκBα/NF-κB Pathway in THP-1 Cells

NF-κB is a key transcription factor in the IKK2 pathway, and may activate the IL-8 promoter to induce gene transcription [21]. We next investigated whether the IKK2/IκBα/NF-κB pathway was involved in the saliva-induced upregulation of IL-8 mRNA levels in THP-1 cells. The blocking of IKK2 by BMS-345541 (25 μM) or NF-κB by BAY 11-7082 (12 μM) significantly attenuated the increase in IL-8 mRNA levels in saliva-treated THP-1 cells (Figure 3A). These data indicate that the saliva from patients with periodontal disease induced the IL-8 promoter via the IKK2/IκBα/NF-κB pathway in THP-1 cells (Figure 3B).

### 2.6. Induction of Heme Oxygenase-1 (HO-1) and Inhibition of IL-8 Expression by CAPE in THP-1 Cells

**Figure 4:**
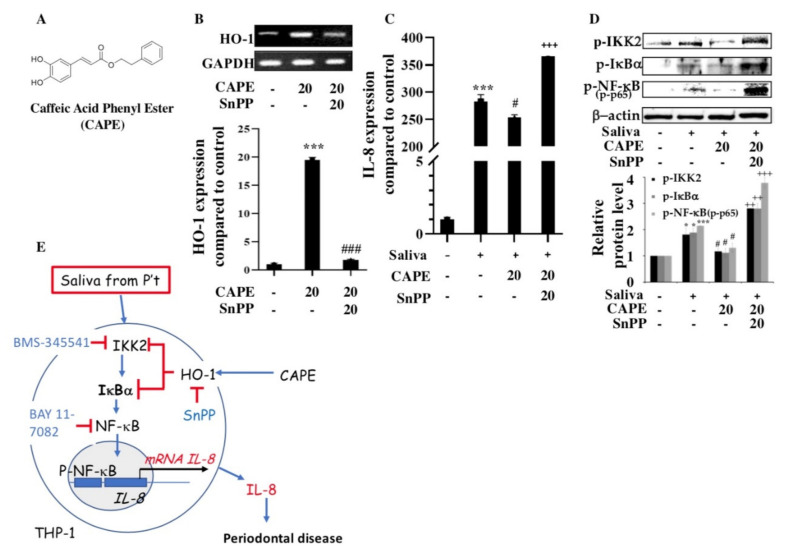
The effects of caffeic acid phenethyl ester (CAPE) on saliva-induced interleukin-8 (IL-8) expression in THP-1 cells and potential underlying molecular mechanisms. (**A**) Structure of CAPE. (**B**) Effects of CAPE (20 μM) and tin-protoporphyrin (SnPP, heme oxygenase-1 (HO-1) inhibitor; 20 μM) on HO-1 mRNA and protein expression in THP-1 cells. Data represent the mean ± standard error of the mean (SEM; *n* = 3). *** *p* < 0.001 compared to control group; ^###^ *p* < 0.01 compared to CAPE-treated group (Student’s *t*-test). (**C**) Effects of CAPE and SnPP on saliva-induced IL-8 mRNA expression in THP-1 cells. (**D**) Levels of phosphorylated IKK2, IκBα, and NF-κB (p65) proteins in THP-1 cells stimulated with 5% saliva alone or pretreated with 20 μM CAPE or CAPE combined with SnPP before saliva treatment. Data represent the mean ± SEM (*n* = 3). * *p* < 0.05, *** *p* < 0.001 compared to control group; ^#^ *p* <0.05 compared to saliva-treated group; ^++^ *p* < 0.01, ^+++^ *p* < 0.001 compared to CAPE-treated group (Student’s *t*-test). (**E**) Working model for saliva-induced IL-8 expression via the IKK2/IκBα/NF-κB pathway in periodontal disease. Blue arrow = stimulation; red line with an end bar = inhibition; black arrow = direction or flow.

We also demonstrated that CAPE (Figure 4A) increased HO-1 mRNA and protein expression in THP-1 cells, whereas tin-protoporphyrin (SnPP, 20 μM), the HO-1 inhibitor, almost completely abolished these effects (Figure 4B). Furthermore, primary macrophages showed a massive inflammatory response, reflected by IL-8 expression, upon exposure to human saliva from patients with periodontitis. In the presence of CAPE, IL-8 expression was almost completely abolished (Figure 4C). However, SnPP reversed the anti-inflammatory effect of CAPE on saliva-induced THP-1 cells (Figure 4C). Moreover, CAPE inhibited the activation of IκBα and NF-κB in saliva-induced THP-1 cells, whereas blocking HO-1 activity with SnPP reversed the inhibition of IKK2, IκBα, and NF-κB phosphorylation by CAPE (Figure 4D).

## 3. Discussion

Chronic inflammation in periodontitis is accompanied by oxidative stress and the overexpression of proinflammatory cytokines, which together culminate in catabolic events that lead to tissue destruction and tooth loss. The clinical relevance of our data is based on the finding that IL-8 is a potential therapeutic target for treating periodontitis; we also revealed the molecular and cellular mechanisms of the beneficial effects of propolis on oral health. The present study showed that IL-8 was elevated in the saliva of patients with periodontitis compared to that in the saliva of healthy individuals, and thus reducing salivary IL-8 secretion may increase the effectiveness of treatment for periodontal disease. In addition, the treatment of the immune cell line THP-1 with the saliva from patients with periodontitis caused a massive inflammatory response and increased IL-8 expression via the IKK2/IκBα/NF-κB pathway. We demonstrated that CAPE reduced inflammation and suppressed IL-8 expression by inducing HO-1 in THP-1 cells (Figure 4E).

Periodontal diseases are chronic infectious diseases characterized by a destructive inflammatory process, which affects the supporting tissues of the tooth leading to the resorption of the alveolar bone, formation of periodontal pockets, and eventual tooth loss [22]. Interactions between the oral microbiota and the proinflammatory responses of the host have been implicated in the onset and progression of periodontitis. However, the underlying mechanisms of these interactions are unknown. Numerous bacterial species have been isolated from the subgingival plaque, some of which are thought to be closely related to disease onset and progression [23]. Bacteria are the primary etiological factors associated with periodontal disease. Herein, we found that the percentages of *F. nucleatum* and *A. actinomycetemcomitans* in the microbiota of the oral cavity were related to the treatment-associated reduction in salivary IL-8 levels, but not to the effectiveness of the periodontitis treatment. It was previously shown that IL-8 levels were significantly upregulated (by 16–33-fold) by *F. nucleatum* [24]. In our previous study, baseline salivary IL-8 levels showed a significant positive correlation with those of the other two proinflammatory cytokines, IL-6 and TNF-α (*r* = 0.39–0.55). The inflammatory response and disease severity, based on the periodontal clinical parameters, were positively correlated with salivary IL-8 levels. Recent studies showed that plasma IL-8 levels were similar between healthy subjects and patients with periodontitis. However, IL-8 levels were higher in patients who had both periodontitis and diabetes than in periodontally healthy subjects or patients with periodontitis without concomitant diabetes [25].

Periodontal disease is characterized by inflammatory cell accumulation in the extravascular gingival connective tissue [26]. Cytokines perform an important role in the pathogenesis of chronic inflammatory diseases. Due to their proinflammatory and neutrophil chemotactic properties, cytokines such as interleukins may play a central role in the pathogenesis of periodontitis. We detected high levels of IL-8 in the saliva of patients with periodontitis, and other studies have also suggested that IL-8 plays a significant role in the pathogenesis of periodontitis [27]. It is likely that locally secreted IL-8 induces neutrophil extravasation at the site of inflammation, and that the numerous neutrophils present in the lamina propria and in the epithelium of inflamed gingiva are directed there by IL-8. In addition, IL-8 may attract T cells and induce the motility of CD45RO^+^ γδ and γδ T cells present in inflamed gingiva [28,29]. Moreover, IL-8 plays an important role in the pathology of chronic inflammatory diseases and is secreted by a variety of cells, including monocytes, fibroblasts, lymphocytes, and endothelial cells. In inflamed gingival tissues, IL-8 was expressed in epithelial cells and macrophages. Therefore, IL-8 levels in the gingival crevicular fluid are valuable for detecting inflammation in the periodontal tissue [30].

A previous study reported that IKK2 is a target for the treatment of inflammation-associated bone loss [31]. The IKK2 subunit is an inflammatory signal activator, which phosphorylates IκBα and targets it for the release of NF-κB (p65). The pivotal role of the transcription factor NF-κB in regulating inflammation is well known [32]. The NF-κB protein complex controls the expression of cytokines, such as IL-8 and TNF-α, which are important mediators of inflammation. IKK2 but not IKK1 is essential for inflammation-induced bone loss and is required for osteoclastogenesis in vivo [33]. The specific and selective inhibition of the IKK2 subunit and of the classical NF-κB activation pathway was shown to be an effective approach for treating inflammatory diseases [33,34].

CAPE, a central component of propolis, has been shown to exert anti-inflammatory effects on gingival fibroblasts [35]. A potential treatment strategy to restore periodontal health and remove scaling and root flatness is to reduce inflammation and oxidative stress by local application of CAPE [19,35]. Propolis was shown to improve the periodontal status of patients with type 2 diabetes [36], and CAPE was reported to protect against ligature-induced periodontitis and other detrimental conditions in rats. Thus, CAPE can reduce harmful effects related to inflammation and cytotoxic activity. The results of our study are consistent with previous findings suggesting that the inhibition of the NF-κB subunit p65 by CAPE controls anti-inflammatory activity in periodontitis [37]. Moreover, CAPE suppresses inflammation and oxidative damage considered to cause periodontal disease. Previous studies suggested that HO-1 is a potential target of periodontal therapy [38,39]. Herein, we found that CAPE increased HO-1 expression, thereby inhibiting saliva-induced IL-8 expression. Therefore, CAPE, which can reduce inflammation and suppress IL-8 expression in macrophage/monocyte THP-1 cells, may increase the effectiveness of treatment protocols for periodontal disease. However, further efficacy studies are required before CAPE can be safely used for local treatment in patients with periodontitis.

One limitation of this study is that only patients with periodontitis were recruited, which may explain the lack of any association between the oral microbiota and effectiveness of periodontitis treatment. Periodontitis is a common infectious disease, and patients should have certain quantities of specific pathogens in their oral microbiota to initiate inflammation. Unfortunately, the analysis of oral microbiota composition based on 16S rRNA gene sequencing reflects only the relative abundance of each potential microbial pathogen as a percentage rather than indicating actual levels. Additionally, the association between the oral microbiota and the effectiveness of periodontitis treatment should be evaluated in comparison to healthy controls.

## 4. Materials and Methods

### 4.1. Study Participants and Sample Collection

A total of 72 participants were recruited at the Taipei Medical University Hospital under a research protocol approved by the Research Ethics Committee of the Taipei Medical University Joint Institutional Review Board N201802045 (Taipei, Taiwan). The study was conducted in accordance with the Helsinki Declaration of 1975, as revised in 2013. Informed consent was obtained from all study participants. Those enrolled were eligible for the comprehensive periodontal treatment project and for non-surgical periodontitis treatment on their first visit, and satisfied the clinical criteria for periodontal disease as mandated by the National Health Insurance Administration, Ministry of Health and Welfare, Taiwan [40]. Subjects with a history of periodontal therapy, cancer, chronic diseases like diabetes and hypertension, and smoking, as well as pregnant women, were excluded from this study. All clinical examinations and treatments were carried out by a periodontist. The detailed protocols for measuring the clinical parameters and collecting saliva are described in our previous study [41]. The clinical parameters of periodontal disease, which included the PI (oral hygiene index), BOP (inflammatory index), and PPD (disease severity index), were recorded at baseline and after clinical treatment. The PI was calculated by dividing the number of plaque-containing surfaces by the total number of available surfaces, which were measured based on the plaque control record and soft debris and mineralized deposits on the four surfaces (buccal, lingual, mesial, and distal) of each tooth. A periodontal probe inserted into the gingival sulcus at six sites (distobuccal, buccal, mesiobuccal, distolingual, lingual, and mesiolingual) of each tooth was used to measure BOP and the PPD. All periodontal examinations were performed by the same periodontist. The extent of reduction (∆) in PI, BOP, and PPD in each patient, which was calculated by subtracting the respective post-treatment value from that at baseline, was used to evaluate the effectiveness of the periodontal clinical treatment.

Saliva was collected before and after the completion of clinical treatment using the Saliva-Check kit (GC Corporation, Tokyo, Japan). The tubes were stored at −20 °C, and proinflammatory cytokines were analyzed within two months of collection. The concentrations of proinflammatory cytokines (IL-β, IL-6, IL-8, and TNF-α) were determined using a Milliplex MAP human cytokine/chemokine magnetic bead panel kit (Merck Millipore, Darmstadt, Germany). The coefficients of variance for IL-1β, IL-6, IL-8, and TNF-α were 1.01%, 1.62%, 0.61%, and 0.70%, respectively.

### 4.2. Cell Line and Treatment

THP-1 cells (American Type Culture Collection, Rockville, MD, USA) a human promonocytic cell lines were cultured in an RPMI 1640 medium (Gibco BRL, Gaithersburg, MD, USA) supplemented with 10 IU/mL penicillin G, 10 μg/mL streptomycin, 2 mM L-glutamine, and 10% fetal calf serum (HyClone Laboratories, Logan, UT, USA). Briefly, THP-1 cells were differentiated with 50 ng/mL phorbol 12-myristate 13-acetate (Sigma-Aldrich, St. Louis, MO, USA) for three days until >99% of cells were adherent. The cells were pretreated with BMS-345541 (25 μM), NF-κB by BAY 11-7082 (12 μM), or CAPE (20 μM) and tin-protoporphyrin (SnPP, the HO-1 inhibitor; 20 μM) at 37 °C for 1 h, and then treated with a pooled saliva sample obtained from patients with periodontitis for 24 h. All further steps were performed on ice or in a cold room unless otherwise noted.

### 4.3. Subgingival Specimen Collection and Sequencing of the 16S Ribosomal RNA (rRNA) Genes of the Oral Microbiota

The subgingival calculus and surrounding tissues were collected when patients underwent scaling and root planning. DNA was extracted from the subgingival tissues using proteinase K digestion, followed by phenol and chloroform extraction. 16S rRNA genes were amplified using specific primers. All polymerase chain reaction (PCR) amplifications were carried out in a volume of 25 μL containing 0.5 μL of KAPA^®^ high-fidelity PCR master mix (Kapa Biosystems, Wilmington, MA, USA), 0.5 μM forward and reverse primers, and approximately 1 ng of template DNA. Thermocycling conditions were as follows: initial denaturation occurred at 95 °C for 3 min, followed by 30 cycles of denaturation at 95 °C for 30 s, annealing at 57 °C for 30 s, and elongation at 72 °C for 30 s with a final extension at 72 °C for 5 min. The PCR products were mixed with an equal volume of 1× loading buffer containing SYBR Green and subjected to electrophoresis on a 2% agarose gel. Samples with a single bright band between 450 and 500 base pairs were chosen for further experiments. The PCR products were mixed in equal density ratios and then purified using a QIAquick gel extraction kit (Qiagen, Hilden, Germany). Sequencing libraries were generated using the TruSeq nano DNA library prep kit (Illumina, San Diego, CA, USA) following the manufacturer’s recommendations, and index codes were added. Library quality was assessed on a Qubit^®^ 2.0 fluorometer (Thermo Fisher Scientific, Waltham, MA, USA) and Agilent Bioanalyzer 2100 system. The library was then sequenced on the Illumina MiSeq platform to generate 300 base pair paired-end reads. Microbiota raw data were reprocessed, including the merging of reads, primer-trimming, quality-filtration, alignment, and chimera removal, using mothur and QIIME. The levels of 16S rRNA gene reads from four species (*P. gingivalis*, *P. intermedia*, *F. nucleatum*, and *A. actinomycetemcomitans*) in relation to the total rRNA reads were calculated as a percentage for the four species.

### 4.4. Isolation of RNA and Reverse Transcription–PCR

Total RNA was isolated from the THP-1 cells using a TRIzol kit (GibcoBRL, Gaithersburg, MD, USA) according to the manufacturer’s instructions. Reverse transcription was performed using an iScript gDNA Clear cDNA synthesis kit (Bio-Rad, Hercules, CA, USA). Quantitative real-time-PCR analyses were performed using the SensiFAST SYBR kit (BIO-98020, BioLine, Eveleigh, Australia) according to the manufacturer’s instructions. Amplification was performed using a StepOnePlus real-time PCR system (Applied Biosystems, Life Technologies, Carlsbad, CA, USA). The primer sequences are shown in Table S1. Relative gene expression was calculated using the delta CT method. The reactions were performed in duplicate.

### 4.5. Western Blotting

The cells were solubilized in an NETN lysis buffer, and protein levels were measured by using the BAC protein assay (Pierce Biotechnology, Inc., Pittsuburgh, PA, USA). Cell lysates containing sodium dodecyl sulfate buffer and protease inhibitors were separated by sodium dodecyl sulfate–polyacrylamide gel electrophoresis, followed by the transfer of the proteins onto polyvinylidene difluoride membranes. The membranes were blocked, incubated with primary antibodies, and incubated with an appropriate secondary antibody. Protein expression was detected using an enhanced chemiluminescence reagent (Pierce Biotechnology, Inc.). Immunoreactive band intensity was quantified by video densitometry (G-box imaging system, Syngene, Frederick, MD, USA).

### 4.6. Statistical Analysis

All experiments were repeated three times. Data are presented as the mean and standard error of the mean (SEM) of cumulative data from all experiments. Statistical analyses were performed using the Student’s *t*-test. Pearson’s correlation analysis was used to determine the association between salivary cytokines and oral microbiota. All analyses were performed using GraphPad Prism 7.0 software (GraphPad Software, La Jolla, CA, USA). Significance was set at *p* < 0.05.

## 5. Conclusions

We found that IL-8 levels were elevated in the saliva of patients with poor periodontitis treatment. Furthermore, CAPE reduced inflammation by suppressing IL-8 expression in THP-1 cells. These data suggest that IL-8 can serve as a potential biomarker and good therapeutic target, and that CAPE can be used to treat periodontitis. However, preclinical models should be evaluated to determine the effectiveness of CAPE against periodontal inflammation.

## Figures and Tables

**Table 1:** The relationship of baseline clinical parameters with salivary proinflammatory cytokines and representative bacterial species in the oral microbiota of patients (*n* = 72) with periodontal disease.

Characteristics	PI (%)	BOP (%)	PPD (Mean; mm)
Salivary proinflammatory cytokines			
Interleukin-6	0.08	0.08	0.13
Interleukin-8	−0.14	0.45 ***	0.49 ***
Tumor necrosis factor-α	−0.05	0.00	0.04
Selected oral microbiota species			
*Porphyromonas gingivalis*	−0.14	−0.11	−0.03
*Prevotella intermedia*	−0.20	−0.05	−0.06
*Fusobacterium nucleatum*	−0.04	−0.13	−0.08
*Aggregatibacter actinomycetemcomitans*	−0.03	−0.02	−0.10

Data are presented as correlation coefficients. *** *p* < 0.0001; PI: plaque index; BOP: bleeding on probing; PPD: probing pocket depth.

**Table 2:** Relationship of differences between pre- and post-treatment values of clinical parameters with representative bacterial species in the oral microbiota in patients (*n* = 72) with periodontal disease.

Selected Oral Microbiota Species	∆PI (%)	∆BOP (%)	∆PPD (Mean; mm)
*Porphyromonas gingivalis*	0.01 (0.94)	−0.07 (0.55)	−0.001 (0.98)
*Prevotella intermedia*	0.09 (0.44)	−0.19 (0.11)	−0.07 (0.57)
*Fusobacterium nucleatum* subsp. *nucleatum*	0.08 (0.53)	−0.04 (0.72)	−0.02 (0.86)
*Aggregatibacter actinomycetemcomitans*	0.05 (0.69)	−0.03 (0.80)	−0.07 (0.51)

Data are presented as correlation coefficients (*p*-values). PI: plaque index; BOP: bleeding on probing; PPD: probing pocket depth.

## Data Availability

Not applicable.

## Supplementary Materials

The following are available online at https://www.mdpi.com/article/10.3390/ijms22073641/s1, Table S1: Primer sequences.
