# Supplementary material for: IL-8 as a Potential Therapeutic Target for Periodontitis and Its Inhibition by Caffeic Acid Phenethyl Ester In Vitro
Source: Int J Mol Sci. 2021 Mar 31;22(7):3641. doi: 10.3390/ijms22073641 (PMC8037988; doi:10.3390/ijms22073641)
Supplement: Supplementary file 1 [file ijms-22-03641-s001.pdf]

Supplementary Table 1 (Table S1). Primer sequences

Primer	Sequence_F	Sequence_R
IL-8	ATGACTTCCAAGCTGGCCGTGGCT	TCTCAGCCCTCTTCAAAAACTTCTC
HO-1	AAGACTGCGTTCCTGCTCAAC	AAAGCCCTACAGCAACTGTCG
GAPDH	GTCTCCTCTGACTTCAACAGCG	ACCACCCTGTTGCTGTAGCCAA
